# The dual glucose-dependent insulinotropic polypeptide (GIP) and glucagon-like peptide-1 (GLP-1) receptor agonist tirzepatide: a novel cardiometabolic therapeutic prospect

**DOI:** 10.1186/s12933-021-01412-5

**Published:** 2021-11-24

**Authors:** Enrique Z. Fisman, Alexander Tenenbaum

**Affiliations:** Department of Cardiology, Sackler Faculty of Medicine, Tel Aviv University, 6997801 Ramat Aviv, Israel

**Keywords:** Dual GLP-1/GIP receptor agonist, Glucagon-like peptide-1, Glucose-dependent insulinotropic polypeptide, Incretins, Obesity, Tirzepatide, Triglycerides, Type 2 diabetes mellitus

## Abstract

Incretin hormones are peptides released in the intestine in response to the presence of nutrients in its lumen. The main incretins are glucagon-like peptide-1 (GLP-1) and glucose-dependent insulinotropic polypeptide (GIP). GLP-1 stimulates insulin secretion, inhibits glucagon secretion at pancreatic α cells and has also extrapancreatic influences as slowing of gastric emptying which increases the feeling of satiety. GIP is the main incretin hormone in healthy people, causative of most the incretin effects, but the insulin response after GIP secretion in type 2 diabetes mellitus (T2DM) is strongly reduced. Therefore, in the past GIP has been considered an unappealing therapeutic target for T2DM. This conception has been changing during recent years, since it has been reported that resistance to GIP can be reversed and its effectiveness restored by improving glycemic control. This fact paved the way for the development of a GIP receptor agonist-based therapy for T2DM, looking also for the possibility of finding a combined GLP-1/GIP receptor agonist. In this framework, the novel dual GIP and GLP-1 receptor agonist tirzepatide seems to be not just a new antidiabetic medication. Administered as a subcutaneous weekly injection, it is a manifold single pharmacological agent that has the ability to significantly lower glucose levels, as well as improve insulin sensitivity, reduce weight and amend dyslipidemia favorably modifying the lipid profile. Tirzepatide and additional dual GLP-1/GIP receptor agonists that could eventually be developed in the future seem to be a promising furthest advance for the management of several cardiometabolic settings. Obviously, it is too early to be overly hopeful since it is still necessary to determine the long-term effects of these compounds and properly verify the potential cardiovascular benefits. Anyway, we are currently facing a novel and very appealing therapeutic option.

## Background

Incretin hormones are peptides released in the intestine in response to the presence of nutrients in its lumen. The observation that the induced effect on insulin secretion is different when oral glucose was administered versus the same amount of glucose parenterally coined the term ‘incretin effect’, supporting the idea that some substances secreted in the intestines favored the release of insulin after ingestion [1]. This effect is part of the entero-insular axis of glucose homeostasis and is estimated to be responsible for at least 50% of insulin secretion [2, 3].

The main incretins are glucagon-like peptide-1 (GLP-1) and glucose-dependent insulinotropic polypeptide (GIP). GLP-1 is a 30 amino acid peptide produced by the L cells, enteroendocrine cells of the distal ileum and colon, and GIP is a 4 amino acid peptide produced by the K cells of the duodenum and jejunum. Both hormones are rapidly released after ingestion, apparently under neural control, and stimulate insulin production in pancreatic β cells in a glucose-dependent manner. Furthermore, GLP-1 decreases glucagon secretion from pancreatic α cells and has also extrapancreatic influences as a direct suppressive effect on appetite centers and a slowing of gastric emptying increasing the feeling of satiety. It has been shown that in patients with type 2 diabetes mellitus (T2DM) the response to the incretin effect is altered as a result of a severe defect in the sensitivity for GIP in the β cells [4] and a reduction in food-induced GLP-1 secretion [5, 6].

Plasma concentrations of GLP-1 and GIP are very low in the fasting state and increase 15–30 min after ingestion. The incretin effect is very brief because it remains active 1–2 min after its secretion and is then inactivated by the enzyme dipeptidylpeptidase-4 (DPP-4) [7]. This very short action limited the initial enthusiasm for the possible usefulness of incretins for the treatment of T2DM, and in turn stimulated the development of both GLP1 agonists resistant to DPP-4, with a longer half-life and of DPP-4 inhibitors that prolong the half-life of native incretins. A large real world evidence demonstrated that when comparing several GLP-1 receptor agonists versus DPP-4 inhibitors treatments in patients with a baseline cardiovascular risk, the incidence of adverse events was much lower for the former [8]; the GLP-1 semaglutide reduced the risk of major adverse cardiovascular events vs comparators in a broad T2DM population [9].

It should be pinpointed that while GIP acts almost exclusively at the pancreatic β level, there are widely distributed receptors for GLP-1 (R-GLP1) in the endocrine pancreas (α and β), in heart, stomach, adipose tissue, vagus nerve and in various regions of the central nervous system. Animal studies have shown that GLP-1 promotes neogenesis and cell proliferation, inhibits apoptosis, and increases the mass of β cells [6]. On the other hand, a series of positive effects have been described in pathways involved in vascular atherogenesis, endothelial function [7, 10] and ventricular contractility [11]. Thus, in addition to regulating glucose control, protective effects are attributed to this incretin at the insular, neural and cardiovascular level.

GIP is the main incretin hormone in healthy people, causative of most the incretin effects but the insulin response after GIP secretion in T2DM is reduced [12]. It has been reported that there is no reduction in its secretion in patients with T2DM [13] but a nearly total loss of insulinotropic effect is observed, even at supraphysiological concentrations, implying the existence of GIP resistance [14]. Therefore, in the past GIP has been considered an unappealing therapeutic target for T2DM. This conception has been changing during recent years, since it has been reported that resistance to GIP can be reversed and its effectiveness restored by improving glycemic control [14]. A potential additional advantage of GIP is the protection against hypoglycemia since GIP infusion is accompanied by a decreased need for further glucose administration to maintain a satisfactory glycemic level during an insulin-induced hypoglycemic clamp [15]. Moreover, it improves triglyceride clearance and the sensitivity of adipose tissue to insulin, which may prevent ectopic fat deposition [16]. These facts paved the way for the development of a GIP receptor agonist-based therapy for T2DM, looking also for the possibility of finding a dual GLP-1/GIP receptor agonist [17].

### A dual GLP-1/GIP receptor agonist

**Fig. 1:**
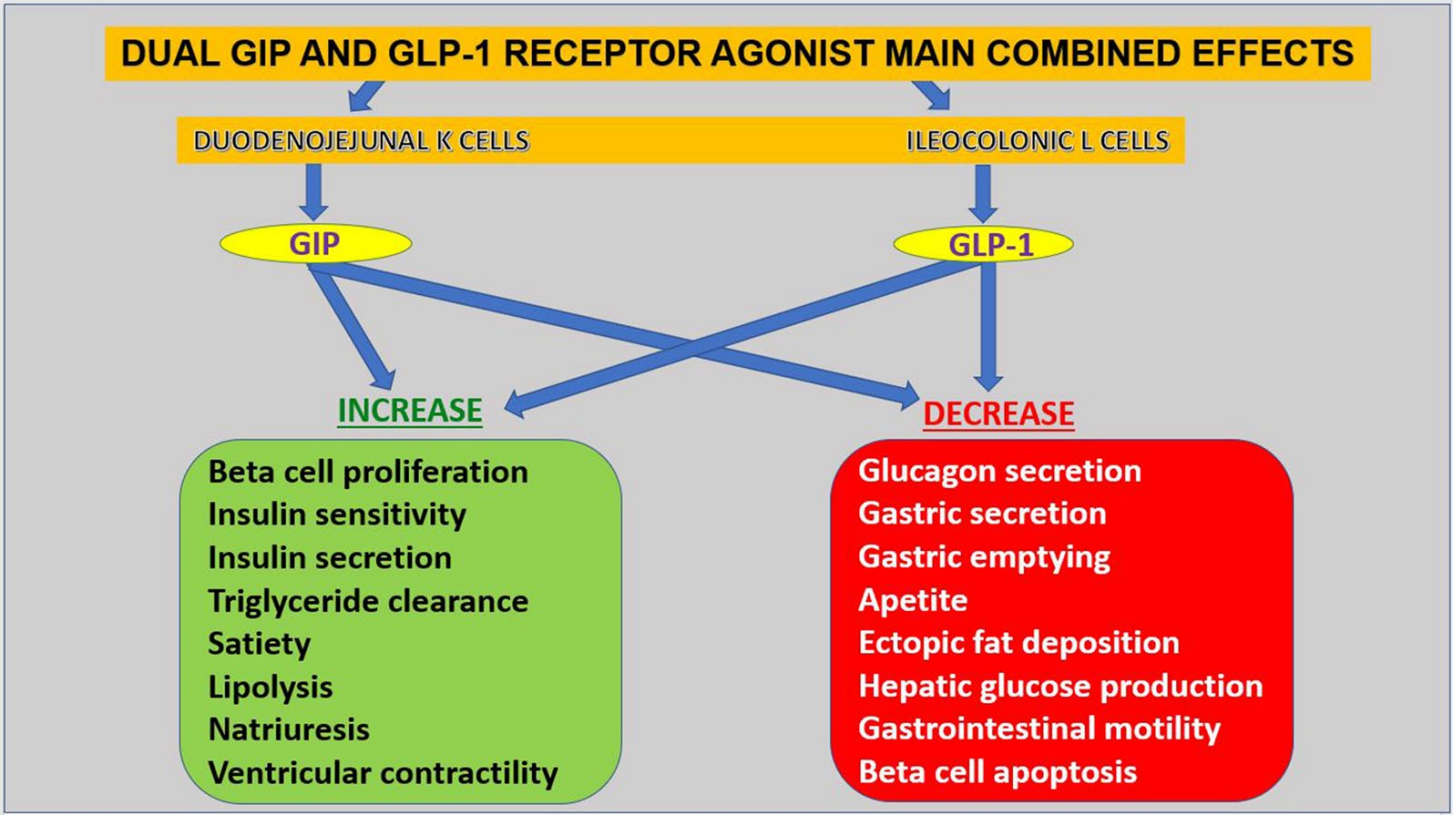
Main physiological actions of the dual GIP and GLP-1 agonist tirzepatide. GIP—glucose-dependent insulinotropic polypeptide; GLP-1—glucagon-like peptide-1. Despite the similarities between the two incretins, it should be pinpointed that while GIP activity is mainly pancreatic, GLP-1 activity is systemic

An ideal antidiabetic medication should present proven efficacy in lowering elevated glucose levels, promote weight loss, have low risk of hypoglycemia and offer cardiovascular benefits [18]. The idea of simultaneously activating both the GIP and GLP-1 receptors seems appealing for treatment of T2DM since it may significantly boost insulin secretion and improve insulin sensitivity. The main physiological actions of such compound are depicted in Fig. 1; the rationale is based on the fact that improved glycemia restores sensitivity to GIP [14, 16] and peptide engineering enables the design of hybrid ligands exhibiting dual agonism, as proved in experimental studies [19].

Tirzepatide, the first dual GIP and GLP-1 receptor agonist, is in keeping with these conceptions. Its chemical formula is based on the GIP amino acid sequence and its half-life of approximately 5 days is compatible with a once-weekly subcutaneous administration. Chemically, tirzepatide is a synthetic linear peptide containing 39 aminoacids based on the native GIP sequence. This basic structure is accompanied by a 20-carbon fatty diacid moiety that prolongs its half-life. The mechanism of action is very imbalanced, since while it has a comparable GIP receptor binding affinity to native GIP, the affinity to the native GLP-1 receptor is five times lower [20, 21]. So, it is a bireceptor agonist, product of the above mentioned peptide engineering, created as a single agent possessing activity at more than one pharmacological target.

The drug was designed for once-weekly subcutaneous administration and early clinical investigation of tirzepatide demonstrated exceptional efficacy for glucose lowering and weight loss in T2DM. A phase 1 proof-of-concept clinical trial was conducted in 53 people with T2DM and translated the favorable preclinical data into clinical facts; the compound delivered clinically meaningful improvement in glycemic control and body weight warranting further clinical evaluation for the treatment of T2DM and obesity [21]. About 30% of patients receiving a 15 mg dose reached normoglycemia with hemoglobin A1C (HbA1C) < 5.7% and 25% of patients lost ≥ 15% of their body weight in a 26-week phase 2b trial [22, 23].

Compared to the GLP-1 agonist dulaglutide, tirzepatide reduced HbA1C by 1.6%, 2.0%, and 2.4% in the 5, 10, and 15 mg dose groups, respectively, compared with 1.1% only for dulaglutide 1.5 mg. Moreover, 8% of patients receiving 10 mg and 30% dosed with 15 mg reached normoglycemia (HbA1C < 5.7%) compared with 2% of subjects treated with dulaglutide. Tirzepatide at 5 mg and 10 mg provided superior glycemic and bodyweight control versus dulaglutide, presenting similar tolerability [22]. These beneficial effects were confirmed in the SURPASS-1 study, the first randomized controlled phase 3 trial of tirzepatide. Study participants had a mean duration of diabetes of 4.7 years, a baseline A1C of 7.9% and a baseline weight of 85.9 kg. Nearly 90% of all participants taking tirzepatide achieved the standard HbA1C goal of > 7% and more than half taking the highest of the three doses also achieved an HbA1C > 5.7%, a level observable in people without diabetes. No events of severe hypoglycemia (< 54 mg/dL) were observed. Regarding collateral effects, the most commonly reported were gastrointestinal-related (diarrhea, nausea, vomiting, constipation) mainly occurring during the dose escalation period. Thus, these results showed a strong glucose-lowering effects towards a nearly-normal range with weight loss of a not previously reported extent in people with T2DM and without increased risk of clinically significant hypoglycemia [24].

The recently published SURPASS-2 has been conducted in order to compare the efficacy and safety of the three doses of tirzepatide with the GLP-1 agonist semaglutide at an injectable dose of 1 mg in patients with T2DM inadequately controlled with metformin monotherapy; it has been performed in an open-label, 40-week, phase 3 trial [25]. All three tirzepatide doses achieved greater A1C and weight reductions compared to semaglutide. In addition, a composite endpoint comprised of participants who achieved an HbA1C level ≥ 6.5% and weight loss of at least 10%, was assessed. Across the three doses of tirzepatide, all patients achieved this composite endpoint in a significantly greater percent compared to patients on semaglutide, with a satisfactory safety profile [25].

### Effects on lipid profile

T2DM patients have usually atherogenic dyslipidemia, depicted by high triglycerides and low-density lipoprotein cholesterol (LDL-C) levels, accompanied by low levels of high-density lipoprotein cholesterol (HDL-C). The upsurge of very-low-density lipoprotein particles seems to be the core atherogenic dyslipidemia in T2DM, which is partially secondary to insulin resistance [26]. In a study aimed to establish tirzepatde influence on lipid profile [27], at 26 weeks the three dosages of the drug decreased the levels of apoB, apoC-III levels, large triglyceride-rich lipoprotein particles, and small low-density lipoprotein particles compared with both placebo and dulaglutide. Patients with high baseline triglycerides (≥ 150 mg/dL) levels showed a relatively greater reduction in apoC-III compared with those presenting normal levels. The dose-dependent decrease in apoC-III seems to partially explain the reduction in triglycerides following tirzepatide treatment, independently of weight loss. A net improvement in insulin sensitivity and a dose-dependent increase in preheparin plasma levels were also observed [27].

### A novel cardiometabolic therapeutic prospect

Cardiometabolic diseases as diabetes mellitus, heart disease, overweight, obesity, hypertension, hyperlipidemia and its multiple macro and microvascular complications (ischemic cardiomyopathy, stroke, chronic kidney disease, retinopathy, peripheral neuropathy, etc.) are conditions closely related to each other and constitute at present time the leading cause of morbility, disability and death in the world [28].

The molecular and cellular alterations that lead to these diseases begin many years before clinical manifestations become apparent. The development of a manifold single pharmacological agent like tirzepatide that has the ability to significantly lower glucose levels, as well as improve insulin sensitivity, reduce weight and amend dyslipidemia at an early clinical stage is enormously important. Therefore, this compound seems to be not solely a new antidiabetic medication. Tirzepatide, administered as a weekly subcutaneous injection, and additional dual GLP-1/GIP receptor agonists that could eventually be developed in the future seem to be a promising furthest advance for the management of several cardiometabolic settings. Obviously, it is too early to be overly hopeful since it is still necessary to determine the long-term effects of these compounds and properly verify the potential cardiovascular benefits. Anyway, we are currently facing a novel and very appealing therapeutic option.

## Data Availability

Not applicable.

## Abbreviations

DPP-4: Dipeptidylpeptidase-4
GIP: Glucose-dependent insulinotropic polypeptide
GLP-1: Glucagon-like peptide-1
HbA1C: Hemoglobin A1C
HDL-C: High-density lipoprotein cholesterol
LDL-C: Low-density lipoprotein cholesterol
T2DM: Type 2 diabetes mellitus
